# Interplay of sleep patterns and oxidative balance score on total cardiovascular disease risk: Insights from the National Health and Nutrition Examination Survey 2005-2018

**DOI:** 10.7189/jogh.13.04170

**Published:** 2023-12-13

**Authors:** Xiang Chen, Caiyi Wang, Zhitao Dong, Hui Luo, Chunyan Ye, Longyan Li, E Wang

**Affiliations:** 1 Department of Anesthesiology, Xiangya Hospital of Central South University, Changsha, Hunan, China; 2 National Clinical Research Center for Geriatric Disorders, Xiangya Hospital of Central South University, Changsha, Hunan, China; 3 Department of Urology, the Second Xiangya Hospital, Central South University, Changsha, Hunan, China

## Abstract

**Background:**

Unhealthy lifestyle and diet may contribute to the development of cardiovascular disease (CVD), but limited evidence exists regarding the association between sleep patterns, oxidative stress-related exposures to diet and lifestyle, and CVD risk.

**Methods:**

We analysed data from 10 212 adults in the National Health and Nutrition Examination Survey (NHANES) database (2005-2018). Self-report questionnaires were used to collect data on sleep duration, sleepiness, and trouble sleeping, classified into three categories: healthy, intermediate, and poor sleep patterns. Healthy sleep was defined as sleeping seven to nine hours per night with no self-reported sleepiness or trouble sleeping, while intermediate and poor sleep patterns indicated one and two to three sleep problems, respectively. The oxidative balance score (OBS) was calculated based on twenty oxidative stress-related exposures to dietary and lifestyle factors, with a higher score indicating greater antioxidant exposure. Survey-based multivariable-adjusted regression analysis was conducted to examine the association of sleep patterns or OBS alone and combined with the total and specific CVD risk.

**Results:**

Participants with poor sleep patterns had a higher likelihood of developing CVD (odds ratio (OR) = 1.76; 95% confidence interval (CI) = 1.26-2.45, *P* < 0.05), while an inverse association was found between OBS and CVD risk (quartile (Q) 4 vs Q1: OR = 0.67; 95% CI = 0.47-0.94, *P* = 0.02, *P* for trend <0.05). There was an interaction between sleep patterns and OBS (*P* for interaction = 0.03). Participants with unhealthy (intermediate and poor) sleep patterns and pro-oxidant OBS (Q1 and Q2) were significantly associated with increased risk of total CVD (OR = 2.31; 95% CI = 1.42-3.74, *P* < 0.05), as well as angina and congestive heart failure, but not coronary heart disease (CHD). Stratified analysis showed that among individuals without hyperlipidaemia, participants with both unhealthy sleep patterns and pro-oxidant OBS exhibited a higher risk of CHD compared to those with healthy sleep patterns and antioxidative OBS.

**Conclusions:**

Unhealthy sleep patterns and reduced oxidative balance are positively associated with an increased risk of overall and specific CVD. Interventions that target healthy sleep habits and antioxidant-rich diets and lifestyles may be important for reducing the risk of CVD.

Cardiovascular disease (CVD) is a leading cause of morbidity and mortality worldwide [1], accounting for 17.9 million deaths annually, which represents 31% of all global deaths, according to the World Health Organization (WHO). Unhealthy lifestyle factors such as physical inactivity, unhealthy diet, smoking, drinking, and unhealthy sleep patterns are crucial drivers of CVD incidence worldwide [2,3]. Multiple lifestyle factors may alone or interact together to promote CVD development, making it imperative to improve unhealthy lifestyle behaviours to reduce the burden of CVD.

Sleep is essential for maintaining physiological and psychological homeostasis. Poor sleep behaviours may increase the risk of chronic diseases, including CVD, through various underlying mechanisms such as oxidative stress, inflammation, metabolic abnormalities, and sympathetic disturbance [4]. Sleep disorders, such as insomnia or sleep apnea, have been linked to various health problems, including CVD, diabetes, and depression [4,5]. Meanwhile, cross-sectional studies and meta-analyses have revealed a substantial association between abnormal sleep duration, excessive daytime sleepiness, and CVD risk [6,7]. Several studies have also investigated the combined effect of different sleep characteristics on CVD risk [8,9]; however, inconsistent results due to heterogeneity of age groups, sample sizes, sleep patterns, and interactions between sleep characteristics have produced varied findings.

Oxidative stress, caused by an imbalance between antioxidants and oxidative damage, can lead to inflammation and chronic diseases such as CVD and chronic kidney disease [10]. The oxidative balance score (OBS), a novel concept derived from multiple dietary (pro-oxidant and antioxidant nutrients) and lifestyle exposures, including smoking, alcohol consumption, obesity, and physical activity (PA), is a useful tool for evaluating an individual’s oxidative stress status. OBS has been consistently associated with an increased risk of cancer [11], inflammatory diseases [12], and chronic diseases [13], but results for CVD [14,15] risk have been inconsistent, particularly from prospective cohort studies. Incomplete measurement of pro- and antioxidative diets and lifestyle may hinder investigations into CVD and the joint effects with other behaviours, such as sleep patterns.

Given the importance of unhealthy sleep behaviours and oxidative stress in increasing the risk of CVD, it is plausible that sleep patterns and OBS could be co-dependent and influence CVD risk through related pathways. For example, a healthy diet and lifestyle, including a high intake of plant-derived high-fiber foods, increased physical activity, and decreased smoking and alcohol intake, may promote health outcomes through improving metabolic fitness (e.g. reducing insulin resistance), maintaining a stable circadian rhythm and a healthy sleep pattern, and against low-grade subclinical inflammation (neuro-inflammatory phenomena) [16]. Physiologically, sleep deprivation may also affect appetite and decrease motivation for daily PA due to emotional dependency among those behaviours [17]. Beyond their independent health effects, the potential joint effects of these two key behaviours remain largely unknown. We analysed a large and representative United States (US) sample from the National Health and Nutrition Examination Survey (NHANES) 2005-2018 in the present study. Our aim was to investigate whether a combined correlation exists between sleep patterns and OBS with both total and specific CVD and whether the concurrent presence of these factors exerts a more substantial influence on overall and specific CVD than when present alone.

## METHODS

### Study population

NHANES is an ongoing cross-sectional survey focusing on the health and nutritional status of the civilian, noninstitutionalised US population. The nationally representative survey uses a complex, multistage probability design to sample residents from all 50 states and Washington, DC. Participants are randomly approached every two years for household interviews, physical examinations, and laboratory tests. The details of the sampling method and its data collection have been published previously [18]. NHANES is conducted by the National Center for Health Statistics (NCHS), part of the Centers for Disease Control and Prevention (CDC), and all participants provided written informed consent.

This study employed publicly available data from NHANES waves spanning 2005-2006, 2007-2008, 2015-2016, and 2017-2018, without personally identifiable information and according to relevant regulations and guidelines [18,19]. 33 482 participants (aged ≥20 years and <80 years) completed questionnaires and examinations. We excluded 653 women who reported being pregnant during the survey. In addition, we excluded participants with missing sleep, dietary and lifestyle, and chronic disease assessment data. After individuals with missing information were excluded from the data set, a total of 10 212 participants (5301 men and 4911 women) with complete interview and examination data were included in the final analysis.

### Ethics approval

The authors take full responsibility for all aspects of the study and have taken steps to ensure that any questions related to the accuracy or integrity of the work are appropriately investigated and resolved. The research was conducted under the principles outlined in the Declaration of Helsinki. As all information from the NHANES program is publicly available, no medical ethics committee board approval was required. The study protocols for NHANES were approved by the NCHS ethics review board (protocol number 2011-17, https://www.cdc.gov/nchs/nhanes/irba98.htm). Informed consent was obtained from all participants before their involvement in the study.

### Sleep patterns

Sleep duration data were collected using self-report questionnaires (SLD010H) during the health interview. Each participant was asked to answer the computer-assisted personal interview question, “How much sleep do you usually get at night on weekdays or workdays?”. Sleep duration was further categorised in this study as short (six hours or fewer hours per night), normal (seven to nine hours per night), or long (more than nine hours per night) based on previous studies [20,21]. In addition, trouble sleeping was measured by the specific question (SLQ050): “Have you ever told a doctor you had trouble sleeping?”. The responses to “How often do you feel overly sleepy during the day?” (SLQ120) were categorised as usual (less or equal to four times a month) and sleepy (more than four times a month). To generate overall sleep pattern scores, the normal and abnormal factors for sleep behaviour were classified as 0 and 1, respectively. These factors pertained to the sleep mentioned above behaviours and resulted in scores ranging from 0 to 3, which indicated a healthy (point 0), intermediate (point 1), and poor (points 2-3) sleep pattern [22].

### Oxidative balance score

OBS was developed to represent overall oxidative balance based on 16 dietary nutrients and four lifestyle pro-oxidant and antioxidant components [23,24]. Dietary intake data was assessed using the 24-hour dietary recall interviews (24HR) based on the University of Texas Food Intake Analysis System and the US Department of Agriculture Survey Nutrients Database conducted in the Mobile Examination Center. Pro-oxidant factors included total fat, total iron intake, smoking status, alcohol drinking status, and obesity status. Smoking status was divided into three categories: current smoker (if smoking at the time), former smoker (if the participant had smoked ≥100 cigarettes in one’s life but was not a current smoker) or never (if the participant had smoked <100 cigarettes in one’s life) [25]. Antioxidant factors included the intake of dietary fiber, calcium, zinc, copper, selenium, magnesium, vitamin C, vitamin E, vitamin B12, vitamin B6, carotene, riboflavin, niacin, and total folate, as well as PA [24].

For the assignment scheme of OBS components (Table S1 in the **Online Supplementary Document**), each dietary component was categorised into three groups by their sex-specific tertiles. Antioxidants were assigned points on a scale from 0 to 2 for tertile groups 1 to 3, respectively, with higher points indicating increased antioxidant levels. Conversely, pro-oxidants were assigned points inversely, with 0 points for the highest tertile and 2 points for the lowest tertile, reflecting higher pro-oxidant levels. For alcohol drinking status, nondrinkers received 2 points, moderate drinkers with 0 to 15 grams per day (g/d) for female or 0 to 30 g/d for male received 1 point, and heavy drinkers with ≥15 g/d for female or ≥30 g/d for male received 0 points. Obesity scores were assigned based on weight status: obesity (body mass index (BMI)≥30 kg per square meter (kg/m^2^) (2 points), overweight (BMI = 25-30 kg/m^2^) (1 point), and normal weight (BMI<25 kg/m^2^) (0 points). Total PA was calculated as the sum time of walking, moderate and vigorous activity undertaken in a week and was categorised according to the level of the metabolic equivalent of task (MET): low (0-499 MET-minutes activity/week), moderate (500-1000 MET-minutes activity/week), and high (>1000 MET-minutes activity/week) [26]. PA was assigned points based on intensity levels: low-intensity PA (0 points), moderate-intensity PA (1 point), and high-intensity PA (2 points). The overall OBS was calculated by summing the points assigned for each component, ranging from five to 42, with higher scores indicating greater antioxidant exposure. Individuals were divided into quartile groups based on OBS and considered quartile (Q) 1 and Q2 as the pro-oxidant group, while Q3 and Q4 were considered as the antioxidative group.

### Outcome definitions

CVD was determined by a composite of a self-reported physician diagnosis with a standardised health condition or from the medical history questionnaire administered during individual interviews [27]. CVD was assessed by five separate questions (MCQ160B-MCQ160F), including the following: “Has a doctor or other health professional ever told you that you had congestive heart failure (CHF)/coronary heart disease (CHD)/angina/heart attack/stroke?”. A positive response to these questions indicated that the individual was considered positive for CVD. In addition, participants who answered positively to a specific CVD item were categorised as having that particular type of CVD. Participants who refused to answer or responded with “don’t know” were excluded from our analysis due to missing data.

### Demographic and lifestyle characteristics

Demographic data on age, gender, race/ethnicity, family income, education level, marital status, smoking status, and disease status were collected from household interviews using standardised questionnaires. Lifestyle behaviours, including body weight, height, and alcohol intake, were obtained from participants who completed the physical examinations at a mobile examination center. Race was categorised as non-Hispanic white, non-Hispanic black, Mexican American, or other. Family income was categorised as≤US$24 999, US$25 000-54 999, US$55 000-99 999, or≥US$100 000. The family poverty income ratio (PIR) was <1.3, 1.3-3.5, or ≥3.5. Education level was classified as less than high school, high school diploma, or more than high school diploma. Marital status was categorised as married (including married and living with a partner), never married, and separated (including widowed, divorced, and separated). BMI was calculated from participants’ weight and height and classified as normal (<25 kg/m^2^), overweight (25-30 kg/m^2^) and obesity (≥30 kg/m^2^). Diabetes mellitus (DM) was defined as a self-reported doctor diagnosis of DM, glycated hemoglobin A1c (HbA1c)≥6.5%, use of insulin or anti-diabetes drugs, fasting glucose ≥7.0 micromole per liter (mmol/L), random glucose ≥11.1 mmol/L, or oral glucose tolerance test (OGTT)≥11.1 mmol/L [28]. Hyperlipidaemia was defined as total cholesterol ≥200 mg per deciliter (mg/dL), triglycerides ≥150 mg/dL, low-density lipoprotein ≥130 mg/dL, and high-density lipoprotein <40 mg/dL [29]. Alternately, participants who reported using lipid-lowering drugs were also classified as having hyperlipidaemia. Depression was defined as total Patient Health Questionnaire 9 (PHQ-9) scores ≥10 on the self-administered questionnaire.

### Statistical analysis

Sample weights, clusters, and stratification were incorporated into all analyses because of the complex sampling design of the NHANES, as required to analyse the NHANES data [18]. According to the NHANES analytic guidelines, the appropriate survey weight is based on the variable of interest that was collected on the smallest number of respondents. Thus, the sample weight for four cycles of NHANES was calculated by dividing the original two-year sample weight by four and then assigning this weight to each participant. In addition, the primary sampling units (PSU) are identified by the variable representing masked variance pseudo-PSU (SDMVPSU), and the strata from which the PSUs are selected are identified by the variable indicating masked variance pseudo-stratum (SDMVSTRA). These variables were used to accurately estimate the variance.

Categorical variables are presented in the form of frequency and percentage. Differences among quartile groups were compared using the weighted univariate linear regression and the Kruskal-Wallis test for normal continuous and nonnormal continuous variables, respectively. Continuous and categorical variables were compared among different CVD or sleep patterns using the Mann-Whitney U and χ^2^ tests, respectively. Survey-weighted multiple logistic regression analysis was used to examine the association among sleep patterns, OBS and the risk of CVD. OBS was converted to a categorical variable by quartile and *P*-value was computed for trend.

We used three models for covariate adjustments to estimate potential differences in the confounding effects. Model 1 was adjusted for age categories and sex. Model 2 was adjusted for variables in Model 1 plus education, race, marital, PIR, alcohol drinking status, and smoking status, and Model 3 was adjusted for the variables in Model 2 plus BMI categories, physical activity, diabetes, hypertension, hyperlipidaemia, and depression. To analyse possible associations between sleep patterns, OBS, and CVD, we estimated adjusted odds ratios (ORs) and 95% confidence intervals (CIs) for outcomes with poor sleep, intermediate sleep, and normal sleep (as a reference). Moreover, stratified analyses were performed to investigate the association between combined sleep patterns with the OBS group and CHD. All statistical analyses were conducted in R software (version 4.1.2, R Foundation for Statistical Computing, Vienna, Austria) using the package “survey” to account for the complex sampling design. Two-sided *P* < 0.05 was considered statistically significant.

## RESULTS

### Population characteristics

The study included 10 212 participants (4911 females and 5301 males) with a mean age of 45.9 years old, among whom 821 (8.0%) were diagnosed with CVD (**Table 1**). Those diagnosed with CVD were more likely to be male and non-Hispanic white, older, less physically active, and had higher BMI, lower education level, and lower family income. Importantly, participants with CVD tended to receive lower OBS and exhibited poor sleep status, as evidenced by a higher prevalence of trouble sleeping, excessive daytime sleepiness, and abnormal sleep duration.

**Table 1:** Characteristics of non-weighted study participants according to CVD, NHANES 2005 to 2018 (n = 10 212)

Characteristics	Total n (%)	Non-CVD, n (%)	CVD, n (%)	*P*
Age (years)				<0.05*
*20-40*	3928 (38.46)	3874 (41.94)	54 (6.61)	
*41-60*	3659 (35.83)	3417 (40.47)	242 (31.74)	
*61-80*	2625 (25.71)	2100 (17.58)	525 (61.64)	
Gender				<0.05*
*Female*	4911 (48.09)	4603 (49.88)	308 (41.07)	
*Male*	5301 (51.91)	4788 (50.12)	513 (58.93)	
Race				<0.05†
*Non-Hispanic White*	4627 (45.31)	4199 (71.95)	428 (75.45)	
*Non-Hispanic Black*	2161 (21.16)	1964 (9.66)	197 (11.38)	
*Mexican American*	1553 (15.21)	1474 (7.05)	79 (3.35)	
*Other*	1871 (18.32)	1754 (11.35)	117 (9.82)	
BMI				<0.05*
*Normal*	2856 (27.97)	2714 (31.05)	142 (16.30)	
*Overweight*	3383 (33.13)	3128 (32.63)	255 (31.22)	
*Obesity*	3973 (38.91)	3549 (36.32)	424 (52.49)	
Education level				<0.05*
*Less than high school*	1918 (18.78)	1732 (10.87)	186 (14.83)	
*High school diploma*	2373 (23.24)	2144 (23.12)	229 (29.49)	
*More than high school*	5921 (57.98)	5515 (66.01)	406 (55.69)	
Marriage status				<0.05*
*Never married*	1887 (18.48)	1817 (18.11)	70 (7.54)	
*Separated*	1897 (18.58)	1646 (14.83)	251 (26.06)	
*Married*	6428 (62.95)	5928 (67.06)	500 (66.41)	
Family income				<0.05*
*<US$25 000*	2031 (19.89)	1773 (12.62)	258 (21.59)	
*US$25 000-54 999*	3963 (38.81)	3622 (33.01)	341 (40.16)	
*US$55 000-99 999*	2785 (27.27)	2634 (33.89)	151 (23.84)	
*≥US$100 000*	1433 (14.03)	1362 (20.48)	71 (14.41)	
Family PIR				<0.05*
*<1.3*	2611 (25.57)	2329 (15.76)	282 (23.13)	
*1.3-3.5*	3928 (38.46)	3599 (34.41)	329 (38.82)	
*≥3.5*	3673 (35.97)	3463 (49.83)	210 (38.06)	
Alcohol drinking status				<0.05*
*Never*	1370 (13.42)	1299 (16.45)	71 (10.03)	
*Moderate*	1986 (19.45)	1828 (21.25)	158 (19.14)	
*Heavy*	6856 (67.14)	6264 (62.30)	59 2(70.83)	
Smoking status				<0.05*
*Never*	5573 (54.57)	5280 (55.96)	293 (34.01)	
*Current*	2128 (20.84)	1944 (19.40)	184 (23.89)	
*Former*	2511 (24.59)	2167 (24.63)	344 (42.10)	
Physical activity				0.01‡
*Low*	3801 (37.22)	3443 (35.62)	358 (41.93)	
*Moderate*	3003 (29.41)	2746 (30.94)	257 (32.43)	
*High*	3408 (33.37)	3202 (33.44)	206 (25.64)	
Hypertension	3989 (39.06)	3369 (31.90)	620 (71.67)	<0.05*
Hyperlipidemia	6923 (67.79)	6216 (65.87)	707 (88.43)	<0.05*
Diabetes	1598 (15.65)	1279 (10.00)	319 (34.75)	<0.05*
Depression	753 (7.37)	638 (5.92)	115 (12.57)	<0.05*
Trouble sleeping	2602 (25.48)	2250 (26.88)	352 (44.15)	<0.05*
Sleepy	2162 (21.17)	1924 (21.92)	238 (27.74)	0.01‡
Sleep duration				<0.05*
*Normal*	6376 (62.44)	5919 (66.93)	457 (57.03)	
*Short sleep*	3267 (31.99)	2960 (28.46)	307 (33.93)	
*Long sleep*	569 (5.57)	512 (4.61)	57 (9.04)	
Sleep patterns				<0.05*
*Healthy sleep*	4334 (42.44)	4092 (43.60)	242 (29.80)	
*Intermediate sleep*	3628 (35.53)	3340 (35.27)	288 (35.20)	
*Poor sleep*	2250 (22.03)	1959 (21.13)	291 (35.01)	
OBS				<0.05*
Q1	2693 (26.37)	2373 (22.06)	320 (34.46)	
Q2	2580 (25.26)	2372 (24.82)	208 (24.48)	
Q3	2749 (26.92)	2582 (28.83)	167 (22.66)	
Q4	2190 (21.45)	2064 (24.28)	126 (18.40)	

BMI – body mass index, CVD – cardiovascular disease, PIR – poverty-income ratio, OBS – oxidative balance score, Q – quartile

*P < 0.001.

†*P* < 0.01.

‡*P* < 0.05.

Regarding sleep patterns and OBS, poor sleep was more commonly observed among female and white participants who were older, had lower income, and had higher BMI, which is similar to most CVD participants. Conversely, participants with higher OBS quartiles had lower BMI, higher income, and received more education, which suggests that they were less likely to experience poor sleep and develop CVD (Tables S2 and S3 in the **Online Supplementary Document**).

### Associations between sleep patterns and risk of CVD

After adjusting for all covariates in our full model (model 3), individuals with short sleep duration were found to have a 1.40-fold increased risk of CVD (95% CI = 1.07-1.83, *P* = 0.02) compared to those with normal sleep duration, while no significant difference was observed in long sleep duration. Sleep complaints, including trouble sleeping (OR = 1.47; 95% CI = 1.17-1.84, *P* < 0.05) and sleepy (OR = 1.36; 95% CI = 1.08-1.71, *P* = 0.01), were significantly associated with CVD compared to individuals without sleep complaints. The relationship between combined sleep patterns (including healthy, intermediate, and poor sleep) and CVD risk is also depicted in **Table 2**. Participants with poor sleep patterns had a higher likelihood of developing CVD (OR = 1.76; 95% CI = 1.26-2.45, *P* < 0.05) when compared to those with healthy sleep patterns. Moreover, participants with poor sleep patterns were associated with a higher possibility of CHF (OR = 2.68; 95% CI = 1.66-4.32, *P* < 0.05), angina (OR = 3.18; 95% CI = 1.93-5.23, *P* < 0.05), heart attack (OR = 1.94; 95% CI = 1.16-3.24, *P* = 0.01), and stroke (OR = 1.93; 95% CI = 1.19-3.13, *P* < 0.05), but not CHD (OR = 1.43; 95% CI = 0.933-2.19, *P* = 0.10) (Table S4 in the **Online Supplementary Document**).

**Table 2:** Weighted odds ratios with 95% CI for the associations between sleep patterns and CVD

Characteristics	Crude model, OR (95% CI)	Model 1, OR (95% CI)*	Model 2, OR (95% CI)†	Model 3, OR (95% CI)‡
Sleep patterns				
*Healthy sleep*	ref.	ref.	ref.	ref.
*Intermediate sleep*	1.46 (1.10, 1.94)	1.51 (1.12, 2.04)	1.43 (1.04, 1.95)	1.37 (1.00, 1.89)
*Poor sleep*	2.43 (1.80, 3.28)	2.62 (1.93, 3.55)	2.27 (1.66, 3.11)	1.76 (1.26, 2.45)
Trouble sleeping				
*No*	ref.	ref.	ref.	ref.
*Yes*	2.15 (1.78, 2.60)	1.81 (1.48, 2.22)	1.76 (1.42, 2.18)	1.47 (1.17, 1.84)
Sleepy				
*No*	ref.	ref.	ref.	ref.
*Yes*	1.37 (1.09, 1.71)	1.77 (1.43, 2.19)	1.58 (1.28, 1.94)	1.36 (1.08, 1.71)
Sleep duration				
*Normal*	ref.	ref.	ref.	ref.
*Short*	1.40 (1.11, 1.77)	1.67 (1.29, 2.15)	1.50 (1.15, 1.95)	1.40 (1.07, 1.83)
*Long*	2.30 (1.49, 3.55)	2.07 (1.36, 3.16)	1.87 (1.15, 3.03)	1.65 (0.98, 2.78)

BMI – body mass index, CI – confidence interval, CVD – cardiovascular disease, OR – odds ratio, PIR – poverty income ratio, ref – reference

*Model 1: Adjusted for age categories and gender.

†Model 2: Combination of model 1 and education, race, marital, PIR, alcohol drinking status, and smoking status.

‡Model 3: Combination of model 3 and BMI categories, physical activity, diabetes, hypertension, hyperlipidaemia, and depression.

### Associations between OBS and risk of CVD

**Figure 1:**
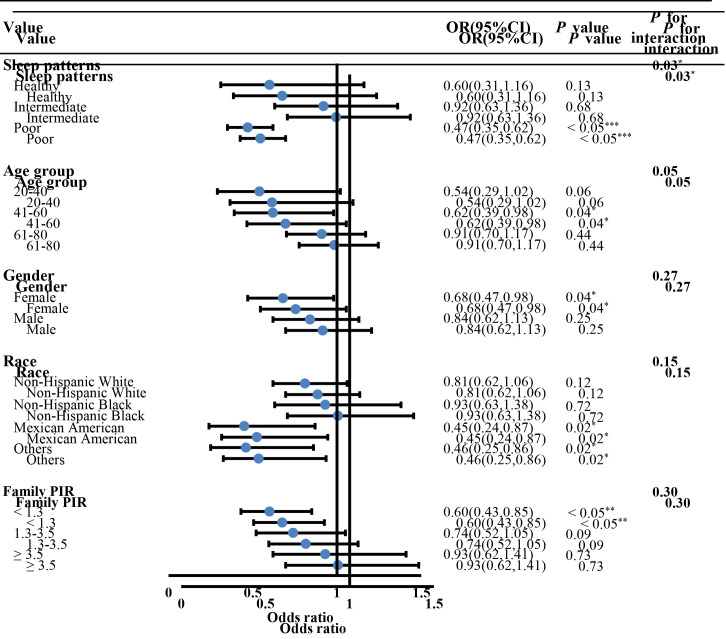
Forest plot of stratified analyses of the associations of antioxidative OBS with CVD compared with pro-oxidant OBS. Adjusted for age (20-40 years, 41-60 years, and 61-80 years), gender (male, female), race (non-Hispanic White, non-Hispanic Black, Mexican American, and others), family PIR (<1.3, 1.3-3.5, and ≥3.5), education levels (less than high school, high school diploma, and more than high school), marital (never married, separated, and married), diabetes, hypertension, hyperlipidaemia and sleep patterns (healthy, intermediate, and poor sleep). The variables examined in this table were not adjusted. OR – odds ratio, CI – confidence interval, CVD – cardiovascular disease, OBS – oxidative balance score, PIR – poverty income ratio. *P*-value: *<0.05, **<0.01, ***<0.001.

An inverse association was observed between OBS and CVD risk (Q4 vs Q1: OR = 0.67; 95% CI = 0.47-0.94, *P* = 0.02, *P* for trend <0.05) (**Table 3**). The negative association was particularly stronger for stroke (Q4 vs Q1: OR = 0.60; 95% CI = 0.40-0.90, *P* = 0.016, *P* for trend <0.05), and CHF (Q4 vs Q1: OR = 0.50; 95% CI = 0.25-0.99, *P* < 0.05, *P* for trend <0.05), but not CHD, angina, and heart attack. Considering that antioxidative OBS has a protective effect on CVD compared with pro-oxidant OBS (OR = 0.71; 95% CI = 0.56-0.90, *P* < 0.05), we focused on the association of antioxidative OBS with CVD by stratified analysis compared with pro-oxidant OBS (**Figure 1**). There was an interaction between OBS and sleep patterns (*P* for interaction = 0.03). In the participants with poor sleep patterns rather than healthy sleep, antioxidative OBS was associated with a lower risk of CVD (OR = 0.47; 95% CI = 0.35-0.62, *P* < 0.05).

**Table 3:** Multivariable weighted odds ratios with 95% CI for the association between OBS and risk of CVD

Characteristics*	OBS Q1	OBS Q2, OR (95% CI)	OBS Q3, OR (95% CI)	OBS Q4, OR (95% CI)	*P* for trend	Pro-oxidative OBS	Antioxidative OBS, OR (95% CI)
CVD	ref.	0.82 (0.58, 1.15)	0.62 (0.44, 0.88)	0.67 (0.47, 0.94)	<0.05	ref.	0.71 (0.56, 0.90)
Congestive heart failure	ref.	0.56 (0.36, 0.88)	0.65 (0.40, 1.06)	0.50 (0.25, 0.99)	<0.05	ref.	0.75 (0.49, 1.16)
Coronary heart disease	ref.	0.90 (0.60, 1.36)	0.67 (0.43, 1.06)	0.75 (0.45, 1.24)	0.14	ref.	0.74 (0.53, 1.05)
Stroke	ref.	0.84 (0.50, 1.42)	0.63 (0.42, 0.92)	0.60 (0.40, 0.90)	<0.05	ref.	0.67 (0.48, 0.94)
Angina	ref.	0.86 (0.49, 1.51)	0.68 (0.36, 1.28)	0.85 (0.38, 1.92)	0.57	ref.	0.82 (0.48, 1.40)
Heart attack	ref.	1.00 (0.61, 1.61)	0.83 (0.51, 1.36)	0.85 (0.48, 1.52)	0.42	ref.	0.84 (0.63, 1.13)

CI – confidence interval, CVD – cardiovascular disease, OBS – oxidative balance score, OR – odds ratio, ref – reference, Q – quartile

*Adjusted for age category, gender, education, race, marital, poverty income ratio, diabetes, hypertension, and hyperlipidaemia.

### Associations between combined sleep patterns and OBS and risk of CVD

**Figure 2:**
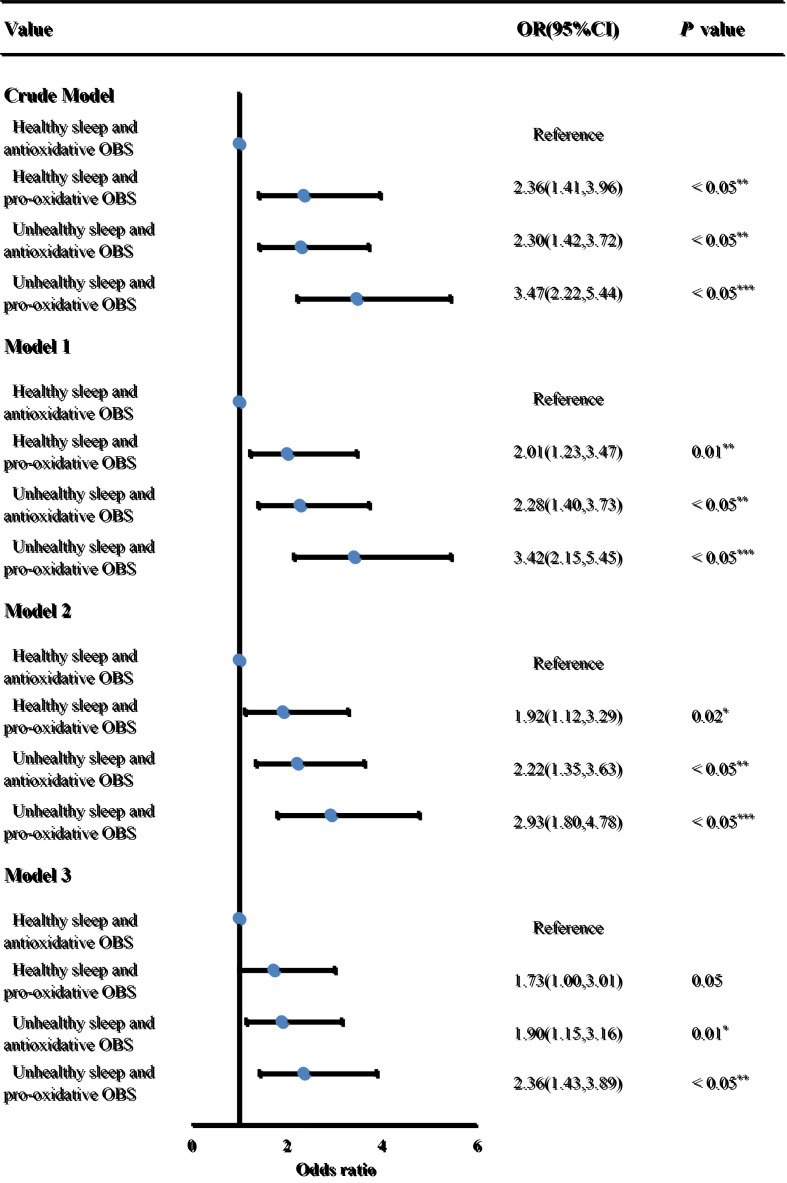
Logistic regression analyses of the association between combined sleep patterns and OBS and CVD. Adjusted for model 1: age categories and gender; model 2: model 1, education levels, race, marital, and PIR; model 3: model 2, diabetes, hypertension, hyperlipidaemia, and depression. CI – confidence interval, OBS – oxidative balance score, OR – odds ratio. *P*-value: *<0.05, **<0.01, ***<0.001.

We divided participants into four sub-groups based on the interaction effects: healthy sleep pattern and antioxidative OBS, healthy sleep pattern and pro-oxidant OBS, unhealthy sleep patterns and antioxidative OBS, and unhealthy sleep patterns and pro-oxidant OBS (**Figure 2**). Participants with unhealthy sleep patterns (intermediate and poor sleep) and pro-oxidant OBS were found to have a higher risk of overall CVD when compared to those with healthy sleep and antioxidative OBS (OR = 2.31; 95% CI = 1.42-3.74, *P* < 0.05). The association between combined unhealthy sleep patterns and pro-oxidant OBS and CVD risk was particularly stronger for CHF (OR = 5.17; 95% CI = 1.73-15.49, *P* < 0.05) and angina (OR = 4.04, 95% CI = 1.71-9.55, *P* < 0.05), followed by heart attack (OR = 2.52; 95% CI = 1.14-5.56, *P* = 0.02), and stroke (OR = 2.20; 95% CI = 1.25-3.87, *P* < 0.05), but except for CHD (*P* > 0.05) (Table S5 in the **Online Supplementary Document**).

### Stratified analyses between combined sleep patterns with OBS and CHD risk

Participants without hyperlipidaemia who combined healthy sleep and pro-oxidant OBS had the highest risk of CHD (OR = 35.61; 95% CI = 3.79-334.64, *P* < 0.05), followed by participants with combined unhealthy sleep patterns and pro-oxidant OBS (OR = 16.86, 95% CI = 2.77-102.46, *P* < 0.05), compared to those with healthy sleep and antioxidative OBS, while interactions were not statistically significant (*P* for interaction = 0.08) (**Table 4**).

**Table 4:** OR and 95% CI for the association between the combined sleep patterns with OBS and risk of coronary heart disease stratified by selected factors

Characteristics*	Healthy sleep and antioxidative OBS	Healthy sleep and pro-oxidative OBS, OR (95% CI)	Unhealthy sleep and antioxidative OBS, OR (95% CI)	Unhealthy sleep and pro-oxidative OBS, OR (95% CI)	*P* for interaction
Age (years)					0.44
*20-40*	ref.	1.81 (0.06, 54.60)	0.39 (0.02, 8.28)	1.84 (0.20, 16.81)	
*41-60*	ref.	2.21 (0.44, 11.17)	1.13 (0.31, 4.08)	2.03 (0.49, 8.42)	
*61-80*	ref.	1.69 (0.72, 4.00)	1.93 (0.82, 4.53)	1.74 (0.80, 3.77)	
Gender					0.17
*Female*	ref.	0.83 (0.22, 3.13)	0.51 (0.17, 1.48)	1.22 (0.43, 3.46)	
*Male*	ref.	2.44 (0.86, 6.91)	2.44 (0.95, 6.25)	2.22 (0.88, 5.62)	
Education level					0.31
*Less than high school*	ref.	1.96 (0.62, 6.21)	1.90 (0.65, 5.51)	2.76 (0.91, 8.34)	
*High school diploma*	ref.	1.64 (0.46, 5.88)	0.79 (0.18, 3.50)	0.78 (0.21, 2.94)	
*More than high school*	ref.	1.88 (0.79, 4.48)	2.02 (0.85, 4.78)	2.43 (1.10, 5.40)	
Family PIR					0.75
*<1.3*	ref.	1.64 (0.29, 9.41)	1.74 (0.31, 9.88)	2.43 (0.39, 15.25)	
*1.3-3.5*	ref.	1.63 (0.74, 3.59)	1.31 (0.58, 2.95)	1.33 (0.66, 2.68)	
*≥3.5*	ref.	2.11 (0.55, 8.12)	1.98 (0.66, 5.95)	2.38 (0.75, 7.56)	
Race					0.28
*Non-Hispanic White*	ref.	2.18 (0.79, 6.03)	1.97 (0.77, 5.07)	1.98 (0.85, 4.60)	
*Non-Hispanic Black*	ref.	0.88 (0.23, 3.37)	1.18 (0.32, 4.33)	2.29 (0.63, 8.28)	
*Mexican American*	ref.	2.31 (0.33, 16.08)	1.15 (0.26, 4.97)	1.48 (0.45, 4.87)	
*Other*	ref.	0.63 (0.15, 2.64)	0.46 (0.12, 1.79)	1.27 (0.31, 5.18)	
Diabetes					0.20
*No*	ref.	1.74 (0.70, 4.31)	1.17 (0.44, 3.11)	1.73 (0.74, 4.05)	
*Yes*	ref.	2.30 (0.66, 8.04)	3.30 (1.26, 8.63)	2.57 (0.98, 6.71)	
Hypertension					0.35
*No*	ref.	3.17 (0.54, 18.51)	2.40 (0.45, 12.76)	1.76 (0.34, 9.24)	
*Yes*	ref.	1.46 (0.65, 3.30)	1.35 (0.66, 2.75)	1.83 (0.93, 60)	
Hyperlipidaemia					0.08
*No*	ref.	35.61 (3.79, 334.64)	9.64 (1.64, 56.68)	16.86 (2.77, 102.46)	
*Yes*	ref.	1.62 (0.67, 3.92)	1.56 (0.70, 3.49)	1.71 (0.81, 3.61)	

CI – confidence interval, CVD – cardiovascular disease, OBS – oxidative balance score, OR – odds ratio, PIR – poverty income ratio, ref – reference

*Adjusted for age category, gender, education, race, marital, poverty income ratio, diabetes, hypertension, hyperlipidaemia, and depression. The variables examined in this table were not adjusted.

## DISCUSSION

In this nationwide prospective cohort study with up to eight years of NHANES data, we found that combined sleep patterns interacted with OBS, independently or jointly, to affect total CVD risk. Participants with combined unhealthy sleep patterns and pro-oxidant OBS were significantly associated with increased risk of total CVD, as well as specific CVD risk, especially for angina and CHF, except for CHD. Stratified analysis showed that among individuals without hyperlipidaemia, participants with both unhealthy sleep patterns and pro-oxidant OBS exhibited a higher risk of CHD compared to those with healthy sleep and antioxidative OBS.

Previous epidemiological studies have consistently demonstrated that abnormal sleep duration [21,30], insomnia [31], snoring [32], and excessive daytime sleepiness [33] are all risk factors for developing CVD. Studies evaluating other combinations of sleep behaviours have found that individuals with poor sleep patterns are at the highest risk of CVD [34]. Since sleep-related factors are often interrelated, it is essential to assess these combinations. For example, sleep disorders may lead to shorter nighttime sleep duration and excessive daytime sleepiness. Restricting sleep due to long work hours during the day, superimposed on caffeine intake, may lead to poor sleep quality at night and difficulty falling asleep and gradually induce sleep disorders [35]. Although our results indicated that all three sleep patterns increased the overall risk of CVD, poor sleep pattern (at least two sleep problems) was associated with a significantly increased risk of various CVD subtypes, especially angina and CHF. This suggests that these sleep behaviours may act alone through multiple mechanisms that can work together to increase the risk of different CVD subtypes further.

The association between OBS and total CVD risk has been investigated previously, while the results were incongruous. In a national, multicenter, multiracial cohort study in the US comprising 3233 individuals, OBS was calculated by summing up 12 pro-oxidant and antioxidant factors obtained from the diet history questionnaire and lifestyle assessment. The results indicated an inverse association between higher OBS and CVD risk, while not significant in adjusted models [14]. In two prospective cohort studies with median follow-ups of more than 10 years, participants in the higher OBS group (considered more antioxidative) were observed with lower all-cause, all-CVD, and all-cancer mortality risk [36,37]. Although these findings suggest an advantage of antioxidants over pro-oxidative lifestyle exposures, their research focuses on exploring healthy OBS to avoid overall CVD mortality risk, not on the development of CVD. Our finding extends previous work by comprehensively analysing OBS with total CVD and five CVD subtypes risk, searching for more specific correlations. Our results revealed a strong inverse association between OBS and total CVD risk, especially for CHF and stroke, but not for CHD, heart attack, and angina. This may suggest underlying mechanistic differences among various CVD subtypes that existing OBS have not yet identified.

Although previous epidemiological studies have consistently shown that sleep problem, as an unhealthy lifestyle, is closely related to CVD, it remains unclear whether sleep plays a pro-oxidative or antioxidative role in the development of CVD. While unhealthy sleep patterns, including obstructive sleep apnea (OSA) and sleep deprivation, have been suggested to increase oxidative stress [38], recent studies have found that increased inflammatory response may be associated with the apnea-hypopnea index and daytime sleepiness. In contrast, OSA may not be associated with abnormalities in oxidative stress markers without metabolic syndrome [39]. No high-quality research currently supports the impact of specific sleep abnormalities and oxidative balance on CVD risk. However, previous reviews suggest that antioxidant nutrient intake and supplements and a Mediterranean-style diet characterised by abundant plant foods and antioxidant nutrients may protect against cognitive decline and CVD complications in individuals with OSA [40,41]. Therefore, we further explored the combined effects of sleep patterns and OBS on total and specific CVD risk. Our findings suggest that individuals with unhealthy sleep patterns and a pro-oxidative diet and lifestyle were more likely to develop CVD. These results highlight the importance of maintaining healthy sleep behaviours and an antioxidant-rich diet and lifestyle to protect against CVD, even when sleep quality is poor.

Several explanations for the deleterious associations between poor sleep and health have been proposed, although none have been widely confirmed. One possibility is that these associations are not causal but due to residual confounding factors, such as sleep fragmentation, fatigue, or undiagnosed mental disorders like depression [42]. Another hypothesis suggests that unhealthy sleep patterns may alter lifestyle behaviours such as PA, drinking, and smoking, which can affect health outcomes. For example, extended sleep duration may reduce the time available for PA, while poor sleep quality may lead to unhealthy eating habits and obesity, such as increasing the frequency of eating (ie, unhealthy snacks) and changing the timing of intake (ie, eating in the evening or at night). Additionally, excessive smoking and drinking may disrupt normal sleep rhythm, further worsening the impact of abnormal sleep on health. In our study, we found that poor sleep patterns were associated with higher rates of alcohol use, smoking, and obesity in participants with a higher pro-oxidant diet, which collectively represented higher CVD risk. It is important to note that our study used a composite sleep score based on self-reported data, which did not allow us to incorporate the temporal sequence or patterns of sleep and other dietary and lifestyle-related behaviours. Future studies using wearable devices to measure these behaviours will provide further insight into their time-dependent aspects and impact on health.

Our study found that sleep patterns and OBS, independently or jointly, were associated with total CVD and most specific CVD risks, with CHD being an exception. Stratified analyses indicated that the positive association between the combined unhealthy sleep patterns and pro-oxidant OBS and CHD risk was more pronounced among those without pre-existing hyperlipidaemia at baseline. This suggests that interventions focusing on optimal dietary and lifestyle behaviours may be more effective before the onset of hyperlipidaemia for CHD prevention. Hyperlipidaemia is a well-established risk factor for CHD [43]. High circulating lipids, particularly low-density lipoprotein cholesterol, deposited in the coronary arterial wall can cause endothelial cell damage and inflammation, promote plaque formation and rupture, and lead to myocardial ischemia and even necrosis [44]. Previous studies have also shown that poor sleep or pro-oxidant dietary and lifestyle behaviours, including sleep apnea, short sleep duration, unhealthy diet (low in fiber and high in meat), and low PA may lead to CHD caused by abnormal glycolipid metabolism, endothelial dysfunction, oxidative stress, and inflammation [45-48]. Therefore, sleep behaviours, OBS, and hyperlipidaemia may have a synergistic effect on CHD through common pathways, which has also been indicated in our findings that the suggestive interaction correlation was found between the combined unhealthy sleep patterns, pro-oxidant OBS, and hyperlipidaemia.

Several limitations of the present study should be noted. First, although survey-weighted multiple logistic regression analysis adjusted for the covariates, a lack of adjustment for residual and unmeasured confounds could also produce a bias. Second, the cross-sectional nature of our study prevented us from establishing a temporal relationship among sleep, OBS, and CVD; thus, the associations cannot be interpreted as causal relations. There is a possibility that participants may have had a medical condition before the onset of sleep problems. If documentation of unhealthy sleep behaviours and OBS systematically increases the risk of existing CVD being diagnosed, this certainly will inflate the observed associations between them. Third, we relied on self-report questionnaires, which are susceptible to recall and information biases and misclassification of exposures. Although the NHANES data set has been validated in previous research, there is still the potential for data quality issues. For example, unlike general medical conditions, most of the data in NHANES were collected based on interviews and self-questionnaires; the misclassification, misdiagnosis, or under-coding of substance use disorder by the interviewee is possible. Some “control” subjects may suffer from chronic disease despite not having reported any chronic disease diagnosis for some reason in the NHANES; this would weaken the associations observed in this study, making our data susceptible to recall and information biases. Fourth, while the sample size was large, our participants were limited to US residents willing to participate in the study, and the generalisability of our results to the rest of the US and other countries is unknown.

## CONCLUSIONS

Unhealthy sleep patterns and reduced oxidative balance are positively associated with an increased risk of overall and specific CVD. Interventions that target healthy sleep habits and antioxidant-rich diets and lifestyles may be important for reducing the risk of CVD.

## Additional material


Online Supplementary Document
