# Supplementary material for: Interplay of sleep patterns and oxidative balance score on total cardiovascular disease risk: Insights from the National Health and Nutrition Examination Survey 2005-2018
Source: J Glob Health. 2023 Dec 13;13:04170. doi: 10.7189/jogh.13.04170 (PMC10715456; doi:10.7189/jogh.13.04170)
Supplement: Online Supplementary Document [file jogh-13-04170-s001.pdf]

Supplementary Tables

Table S1. Oxidative balance score assignment scheme.

Table S2. Characteristics of non-weighted study participants according to sleep patterns, NHANES 2005 to 2018 (n = 10212).

Table S3. Characteristics of non-weighted study participants according to OBS, NHANES 2005 to 2018 (n = 10212).

Table S4. Weighted odds ratios with 95% confidence intervals for the associations between sleep patterns and specific CVD risk.

Table S5. Weighted odds ratios with 95% confidence intervals for the associations between combined sleep patterns with OBS and specific CVD risk.

Table S1. Oxidative balance score assignment scheme.

OBS components	Property	Male			Female		
		**0**	**1**	**2**	**0**	**1**	**2**
Dietary OBS components							
Dietary fiber (g/d)	A	≤13.20	13.20-20.80	>20.80	≤11.30	11.30-17.30	>17.30
Carotene (RE/d)	A	≤53.58	53.58-176.81	>176.81	≤57.60	57.60-198.53	>198.53
Riboflavin (mg/d)	A	≤1.80	1.80-2.64	>2.64	≤1.40	1.40-2.02	>2.02
Niacin (mg/d)	A	≤22.93	22.93-32.73	>32.73	≤16.29	16.29-23.49	>23.49
Vitamin B6 (mg/d)	A	≤1.74	1.74-2.59	>2.59	≤1.29	1.29-1.89	>1.89
Total folate (mcg/d)	A	≤328.33	328.33-495	>495.00	≤257.39	257.39-385.50	>385.50
Vitamin B12 (mcg/d)	A	≤3.66	3.66-4.40	>4.40	≤2.56	2.56-4.50	>4.50
Vitamin C (mg/d)	A	≤43.23	43.23-104.13	>104.13	≤41.45	41.45-90.35	>90.35
Vitamin E (ATE) (mg/d)	A	≤6.02	6.02-9.57	>9.57	≤5.03	5.03-7.98	>7.98
Calcium (mg/d)	A	≤751.00	751.00-1130.50	>1130.50	≤610.67	610.67-921.50	>921.50
Magnesium (mg/d)	A	≤262.33	262.33-366.50	>366.50	≤211.00	211.00-293.00	>293.00
Zinc (mg/d)	A	≤10.00	10.00-14.65	>14.65	≤7.24	7.24-10.54	>10.54
Copper (mg/d)	A	≤1.08	1.08-1.53	>1.53	≤0.88	0.88-1.25	>1.25
Selenium (mcg/d)	A	≤102.60	102.6-145.48	>145.48	≤74.77	74.77-105.73	>105.73
Total fat (g/d)	P	≤70.92	70.92-104.62	>104.62	≤53.03	53.03-77.44	>77.44
Iron (mg/d)	P	≤12.72	12.72-18.74	>18.74	≤9.79	9.79-14.12	>14.12
Lifestyle OBS components							
Physical activity	A	Low	Moderate	High	Low	Moderate	High
Alcohol (g/d)	P	≥30	0-30	None	≥15	0-15	None
Obesity	P	Obesity	Overweight	Normal	Obesity	Overweight	Normal

Smoking status	P	Current smoker	Former smoker	Never smoker	Current smoker	Former smoker	Never smoker

OBS – oxidative balance score, A – antioxidant; P – prooxidant, RE – retinol equivalent, ATE – alpha-tocopherol equivalent, MET – metabolic equivalent.

Table S2. Characteristics of non-weighted study participants according to sleep patterns, NHANES 2005 to 2018 (n = 10, 212).

**Characteristics**	**Healthy sleep (n = 4334)**	**Intermediate sleep (n = 3628)**	**Poor sleep (n = 2250)**	***P* value**
Age (years)				< 0.05**
20-40	1751(41.99)	1388(39.45)	789(36.05)	
41-60	1429(37.58)	1321(40.24)	909(44.06)	
61-80	1154(20.43)	919(20.30)	552(19.90)	
Gender				0.01*
Female	2009(47.62)	1706(48.73)	1196(53.68)	
Male	2325(52.38)	1922(51.27)	1054(46.32)	
Race				< 0.05***
Non-Hispanic White	1954(72.39)	1588(71.22)	1085(73.22)	
Non-Hispanic Black	731(7.58)	903(11.63)	527(11.02)	
Mexican American	797(8.26)	505(6.26)	251(4.93)	
Others	852(11.77)	632(10.89)	387(10.83)	
BMI				< 0.05***
Normal	1358(33.40)	985(29.69)	513(24.56)	
Overweight	1508(33.49)	1201(33.17)	674(29.73)	
Obesity	1468(33.11)	1442(37.14)	1063(45.71)	
Education levels				< 0.05***
Less than high school	839(11.43)	658(10.86)	421(10.90)	
High school diploma	927(20.52)	891(24.42)	555(27.86)	
More than high school	2568(68.05)	2079(64.73)	1274(61.23)	
Marriage status				< 0.05***
Married	768(17.03)	678(17.17)	441(18.80)	

Separated	664(12.90)	695(15.74)	538(20.22)	
Never married	2902(70.07)	2255(67.08)	1271(60.97)	
Family income				< 0.05***
< US$25 000	747(11.04)	691(12.38)	593(18.59)	
US$25 000-54 999	1704(34.15)	1426(33.30)	833(32.33)	
US$55 000-99 999	1217(33.56)	1022(34.45)	546(30.82)	
⩾ US$100 000	666(21.25)	489(19.88)	278(18.25)	
Family PIR				< 0.05***
< 1.3	992(14.01)	898(15.75)	721(21.22)	
1.3-3.5	1674(34.39)	1449(35.51)	805(33.92)	
≥ 3.5	1668(51.60)	1281(48.74)	724(44.86)	
Alcohol drinking status				0.01*
Never	575(15.71)	501(16.49)	294(16.05)	
Moderate	918(23.50)	667(19.16)	401(19.65)	
Heavy	2841(60.79)	2460(64.35)	1555(64.30)	
Smoking status				< 0.05***
Never	2572(59.53)	1981(55.54)	1020(43.62)	
Current	724(16.08)	773(19.08)	631(27.64)	
Former	1038(24.40)	874(25.38)	599(28.74)	
Physical activity				0.01*
Low	1649(36.59)	1342(36.25)	810(34.49)	
Moderate	1332(32.47)	1050(30.80)	621(28.58)	
High	1353(30.94)	1236(32.95)	819(36.93)	
Hypertension (yes, %)	1477(29.73)	1434(34.48)	1078(43.03)	< 0.05***
Hyperlipidemia (yes, %)	2861(65.02)	2465(68.02)	1597(70.35)	< 0.05**
Diabetes (yes, %)	596(9.75)	528(9.96)	474(17.42)	< 0.05***

Depression (yes, %)	101(1.64)	221(4.90)	431(17.72)	< 0.05***
Congestive heart failure (yes, %)	49(0.65)	85(1.60)	98(2.54)	< 0.05***
Coronary heart disease (yes, %)	117(2.31)	99(2.45)	100(3.90)	0.01*
Angina (yes, %)	48(0.69)	65(1.56)	82(3.08)	< 0.05***
Heart attack (yes, %)	92(1.71)	105(1.91)	118(4.29)	< 0.05***
Stroke (yes, %)	71(1.07)	96(1.80)	105(3.25)	< 0.05***
CVD (yes, %)	242(4.25)	288(6.08)	291(9.71)	
OBS				< 0.05***
Quartile 1	996(19.40)	992(22.98)	705(29.20)	
Quartile 2	1095(24.84)	911(24.63)	574(25.01)	
Quartile 3	1246(30.03)	965(28.50)	538(25.33)	
Quartile 4	997(25.73)	760(23.89)	433(20.45)	

Data were expressed as percentage (frequency%). BMI – body mass index, PIR – poverty-income ratio, CVD – cardiovascular disease, OBS – oxidative balance score. *P*-value: *, < 0.05; **, < 0.01; ***, < 0.001.

Table S3. Characteristics of non-weighted study participants according to OBS, NHANES 2005 to 2018 (n = 10212).

**Characteristics**	**Quartile 1 (n = 2560)**	**Quartile 2 (n = 2588)**	**Quartile 3 (n = 2766)**	**Quartile 4 (n = 2298)**	***P* value**
Age (years)					0.10
20-40	931(39.88)	955(38.17)	1084(40.47)	958(40.51)	
41-60	893(38.74)	909(38.86)	1008(40.30)	849(41.60)	
61-80	736(21.38)	724(22.97)	674(19.22)	491(17.89)	
Gender					0.44
Female	1188(48.49)	1255(51.04)	1338(49.54)	1130(48.19)	
Male	1372(51.51)	1333(48.96)	1428(50.46)	1168(51.81)	
Race					< 0.05***
Non-Hispanic White	1038(67.50)	1116(70.22)	1347(74.60)	1126(75.21)	
Non-Hispanic Black	743(14.82)	587(10.87)	492(7.95)	339(6.47)	
Mexican American	352(6.84)	391(7.02)	407(6.21)	403(7.34)	
Others	427(10.84)	494(11.90)	520(11.25)	430(10.99)	
BMI					< 0.05***
Normal	531(22.47)	692(29.46)	795(29.53)	838(38.09)	
Overweight	812(31.06)	887(33.22)	956(33.84)	728(31.67)	
Obesity	1217(46.47)	1009(37.32)	1015(36.63)	732(30.24)	
Education levels					< 0.05***
Less than high school	648(16.25)	517(11.59)	421(8.89)	332(8.83)	
High school diploma	732(31.23)	599(24.40)	599(21.52)	443(18.35)	
More than high school	1180(52.52)	1472(64.01)	1746(69.59)	1523(72.82)	
Marriage status					< 0.05***
Never married	530(21.25)	457(15.96)	463(15.74)	437(17.73)	
Separated	595(19.08)	481(16.09)	477(14.87)	344(12.64)	

Married	1435(59.67)	1650(67.95)	1826(69.39)	1517(69.64)	
Family income					< 0.05***
< US$25 000	699(19.62)	489(12.78)	464(10.91)	379(10.68)	
US$25 000-54 999	1031(36.41)	1059(35.78)	1044(32.01)	829(30.31)	
US$55 000-99 999	586(28.34)	692(32.51)	829(35.56)	678(35.59)	
⩾ US$100 000	244(15.64)	348(18.94)	429(21.52)	412(23.43)	
Family PIR					< 0.05***
< 1.3	1017(38.33)	1049(36.35)	1019(32.58)	843(32.37)	
1.3-3.5	867(23.48)	648(16.37)	614(13.71)	482(12.74)	
≥ 3.5	676(38.19)	891(47.28)	1133(53.71)	973(54.89)	
Alcohol drinking status					< 0.05**
Never	348(15.10)	340(18.30)	393(15.95)	289(14.81)	
Moderate	450(18.20)	484(19.21)	584(24.32)	468(21.80)	
Heavy	1762(66.70)	1764(62.49)	1789(59.73)	1541(63.39)	
Smoking status					< 0.05***
Never	1101(42.22)	1427(53.58)	1584(56.43)	1461(64.15)	
Current	824(32.74)	518(19.87)	485(15.98)	301(12.63)	
Former	635(25.05)	643(26.55)	697(27.59)	536(23.22)	
Physical activity					< 0.05***
Low	1017(37.97)	895(33.50)	871(31.36)	631(26.65)	
Moderate	779(31.34)	881(35.43)	977(36.92)	762(35.51)	
High	764(30.68)	812(31.06)	918(31.72)	905(37.84)	
Hypertension (yes, %)	1162(38.92)	1039(36.23)	996(32.30)	792(30.90)	< 0.05***
Diabetes (yes, %)	495(13.76)	447(13.28)	389(10.52)	267(9.00)	< 0.05***
Depression (yes, %)	278(11.23)	174(5.54)	177(5.33)	124(4.05)	< 0.05***
Hyperlipidemia (yes, %)	1814(70.09)	1792(69.15)	1865(66.37)	1452(63.99)	< 0.05**

Congestive heart failure (yes, %)	103(2.58)	54(1.28)	41(1.16)	34(0.78)	< 0.05***
Coronary heart disease (yes, %)	107(3.51)	89(3.23)	67(2.15)	53(2.16)	0.04*
Angina (yes, %)	64(2.04)	58(1.72)	40(1.15)	33(1.31)	0.23
Heart attack (yes, %)	119(3.04)	84(2.78)	60(1.96)	52(1.78)	0.06
Stroke (yes, %)	108(2.86)	71(2.15)	54(1.33)	39(1.13)	< 0.05**
CVD (yes, %)	303(8.84)	222(7.03)	165(4.67)	131(4.48)	< 0.05***
Trouble sleeping (yes, %)	720(30.80)	674(29.57)	628(24.82)	580(27.50)	< 0.05***
Sleepy (yes, %)	573(24.75)	518(21.32)	560(20.89)	511(22.71)	0.05*
Sleep duration					< 0.05***
Normal	1410(57.55)	1637(66.86)	1800(69.05)	1529(70.14)	
Short sleep	962(35.07)	809(28.40)	822(26.43)	674(26.55)	
Long sleep	188(7.38)	142(4.74)	144(4.52)	95(3.32)	
Sleep patterns					< 0.05***
Healthy sleep	945(36.59)	1122(42.35)	1258(46.50)	1009(44.10)	
Intermediate sleep	950(35.93)	894(34.78)	968(34.25)	816(36.37)	
Poor sleep	665(27.49)	572(22.87)	540(19.25)	473(19.54)	

Data were expressed as percentage (frequency%). BMI – body mass index, PIR – poverty-income ratio, CVD – cardiovascular disease, OBS – oxidative balance score. *P*-value: *, < 0.05; **, < 0.01; ***, < 0.001.

Table S4. Weighted odds ratios with 95% confidence intervals for the associations between sleep patterns and specific CVD risk.

**Characteristics**	**Angina OR (95% CI)**	**Congestive heart failure OR (95% CI)**	**Coronary heart disease OR (95% CI)**	**Heart attack OR (95% CI)**	**Stroke OR (95% CI)**
Sleep patterns *					
Healthy sleep	Reference	Reference	Reference	Reference	Reference
Intermediate sleep	2.16(1.33, 3.50)	2.27(1.41, 3.64)	1.05(0.64, 1.72)	1.03(0.63, 1.69)	1.43(0.92, 2.21)
Poor sleep	3.18(1.93, 5.23)	2.68(1.66, 4.32)	1.43(0.93, 2.19)	1.94(1.16, 3.24)	1.93(1.19, 3.13)
Trouble sleeping *					
No	Reference	Reference	Reference	Reference	Reference
Yes	1.72(1.15, 2.56)	1.93(1.35, 2.77)	1.21(0.84, 1.75)	1.55(1.05, 2.31)	1.74(1.18, 2.58)
Sleepy *					
No	Reference	Reference	Reference	Reference	Reference
Yes	1.76(1.11, 2.79)	1.74(1.12, 2.70)	1.03(0.75, 1.43)	1.16(0.84, 1.59)	1.53(1.03, 2.28)
Sleep duration *					
Normal	Reference	Reference	Reference	Reference	Reference
Short sleep duration	1.94(1.28, 2.95)	1.44(1.00, 2.08)	1.21(0.87, 1.69)	1.63(1.08, 2.48)	1.38(0.96, 2.00)
Long sleep duration	2.48(1.09, 5.64)	1.83(0.98, 3.44)	1.70(0.69, 4.21)	1.56(0.61, 4.03)	1.26(0.65, 2.45)

OR – odds ratio, CI – confidence interval, CVD – cardiovascular disease, BMI – body mass index, PIR – poverty income ratio.

* Adjusted for age categories, gender, education, race, marital, PIR, alcohol drinking status, smoking status, BMI categories, physical activity, diabetes, hypertension, hyperlipidemia, and depression.

Table S5. Weighted odds ratios with 95% confidence intervals for the associations between combined sleep patterns with OBS and specific CVD risk.

Characteristics	Angina OR (95% CI)	Congestive heart failure OR (95% CI)	Coronary heart disease OR (95% CI)	Heart attack OR (95% CI)	Stroke OR (95% CI)
Sleep patterns and OBS *					
Healthy sleep and antioxidative OBS	Reference	Reference	Reference	Reference	Reference
Healthy sleep and pro-oxidative OBS	1.91(0.67, 5.45)	2.89(0.88, 9.46)	1.83(0.79, 4.24)	2.06(1.01, 4.23)	1.25(0.62, 2.48)
Unhealthy sleep and antioxidative OBS	3.77(1.80, 7.89)	4.77(1.62,14.04)	1.65(0.76, 3.61)	2.47(1.10, 5.56)	1.43(0.79, 2.58)
Unhealthy sleep and pro-oxidative OBS	4.04(1.71, 9.55)	5.17(1.73,15.49)	1.86(0.91, 3.80)	2.52(1.14, 5.56)	2.20(1.25, 3.87)

OR – odds ratio, CI – confidence interval, CVD – cardiovascular disease, OBS – oxidative balance score.

Unhealthy sleep is combined intermediate sleep with poor sleep patterns. Quartile 1 and quartile 2 OBS were considered as the pro-oxidative group, while quartile 3 and quartile 4 OBS were considered as the antioxidative OBS group.

* Adjusted for age category, gender, education, race, marital, family income-to-poverty ratio, diabetes, hypertension, hyperlipidemia, and depression.
